# Supplementary figures and images for: Combinatorial expression of ebony and tan generates body color variation from nymph through adult stages in the cricket, Gryllus bimaculatus
Source: PLoS One. 2023 May 18;18(5):e0285934. doi: 10.1371/journal.pone.0285934 (PMC10194958; doi:10.1371/journal.pone.0285934)

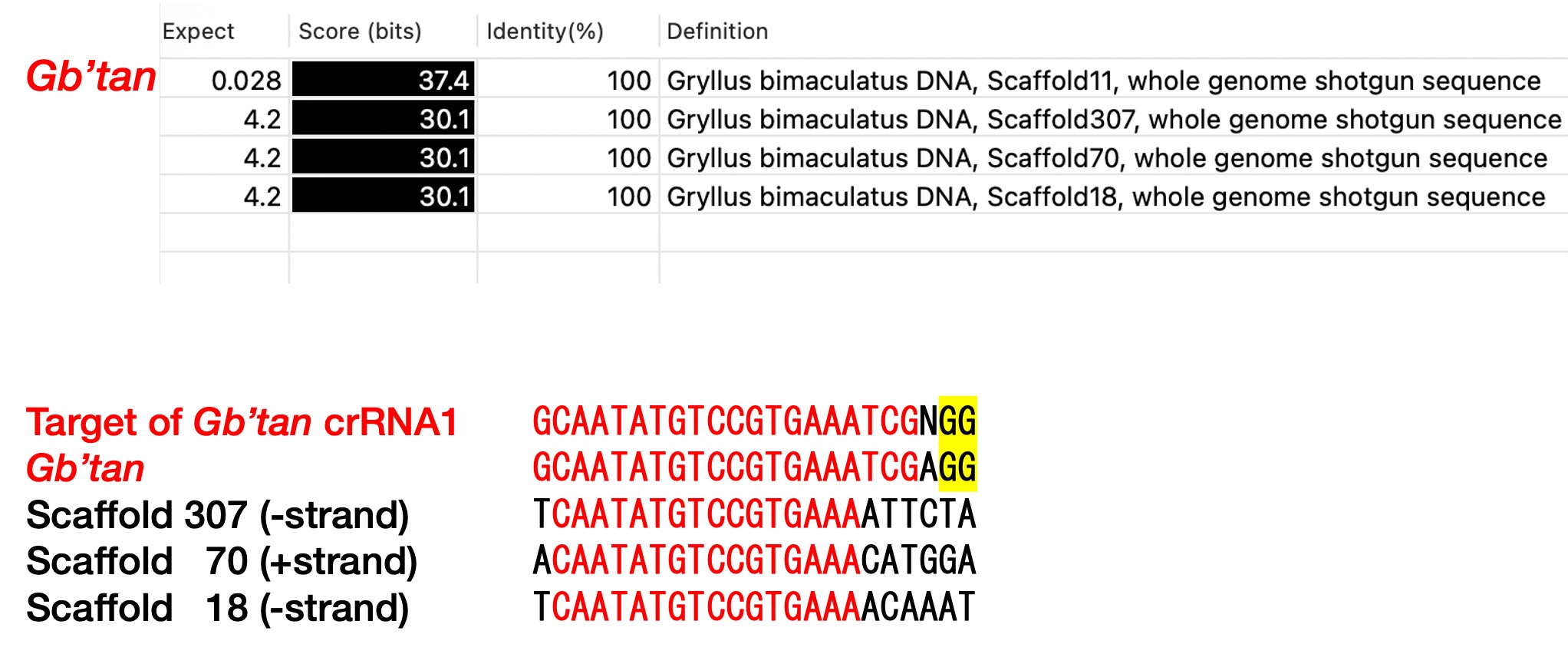

Supplement: S1 Fig — The BLASTN program was used to search for candidates of off-target sequence effects of Gb’tan crRNA1 in the Gryllus genome (GenBank: GCA_017312745.1). In addition to Gb’tan, three other sequences were detected on Scaffold307 (GenBank: BOPP01000307.1), Scaffold70 (GenBank: BOPP01000070.1), and Scaffold18 (GenBank: BOPP01000018.1), but their sequences have no PAM (NGG) sequence. (JPG) [file pone.0285934.s001.jpg]

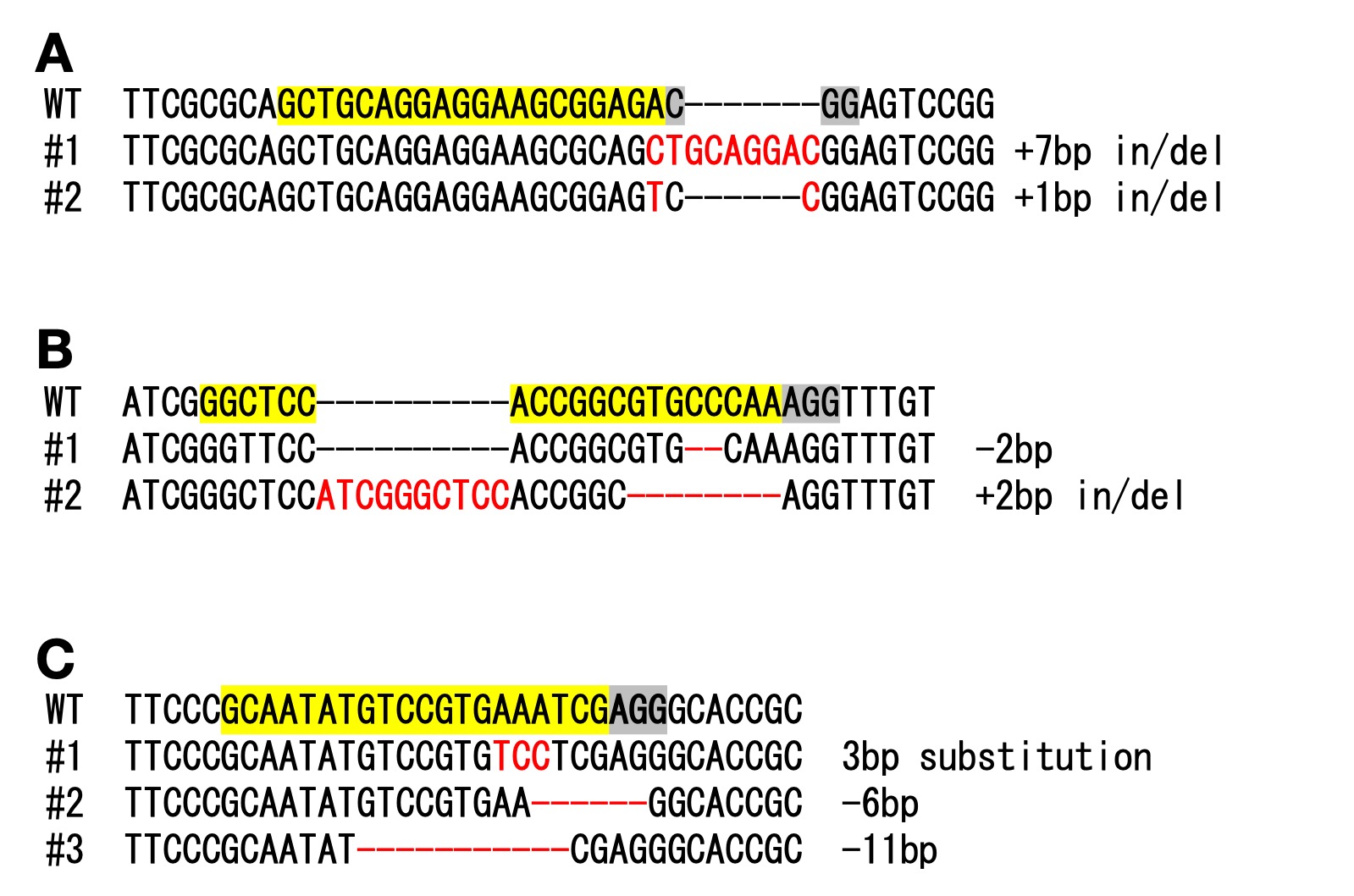

Supplement: S2 Fig — Sequence analysis of mutations introduced around the crRNA target region (highlighted in yellow) in the Gb’ebonycr1 (A), Gb’ebonycr2 (B), and Gb’tancr1 (C) mutants of the G0 generation. (JPG) [file pone.0285934.s002.jpg]

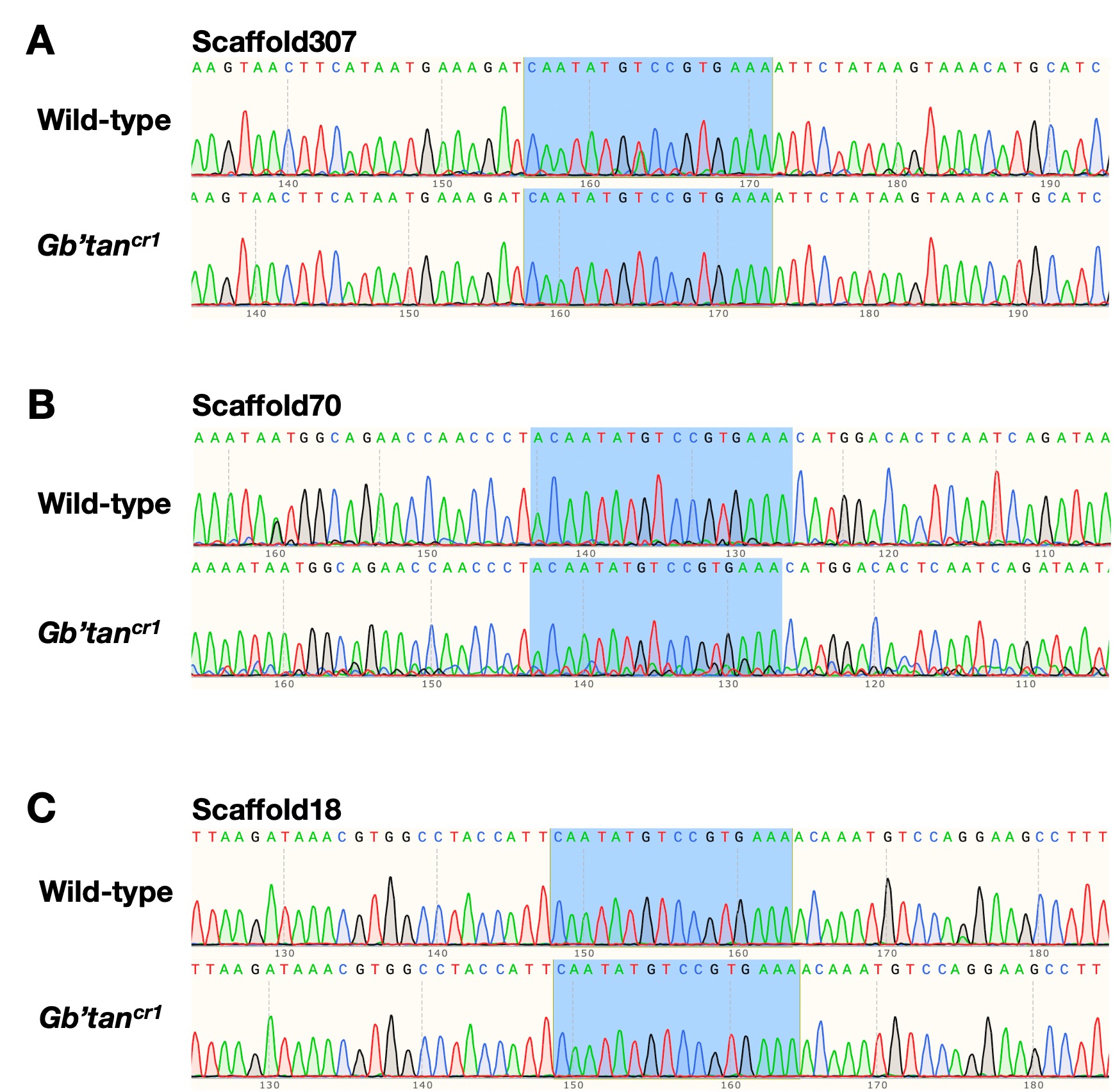

Supplement: S3 Fig — PCR amplification and sequence analysis of off-target sites (highlighted in blue) predicted for Gb’tan crRNA1 ((A) Scaffold307 (GenBank: BOPP01000307.1), (B) Scaffold70 (GenBank: BOPP01000070.1), and (C) Scaffold18 (GenBank: BOPP01000018.1) on G. bimaculatus genomic data (GenBank: GCA_017312745.1)) were performed using the genome of the Gb’tan mutant as a template and the primers listed in S1 Table compared with the wild-type. (JPG) [file pone.0285934.s003.jpg]

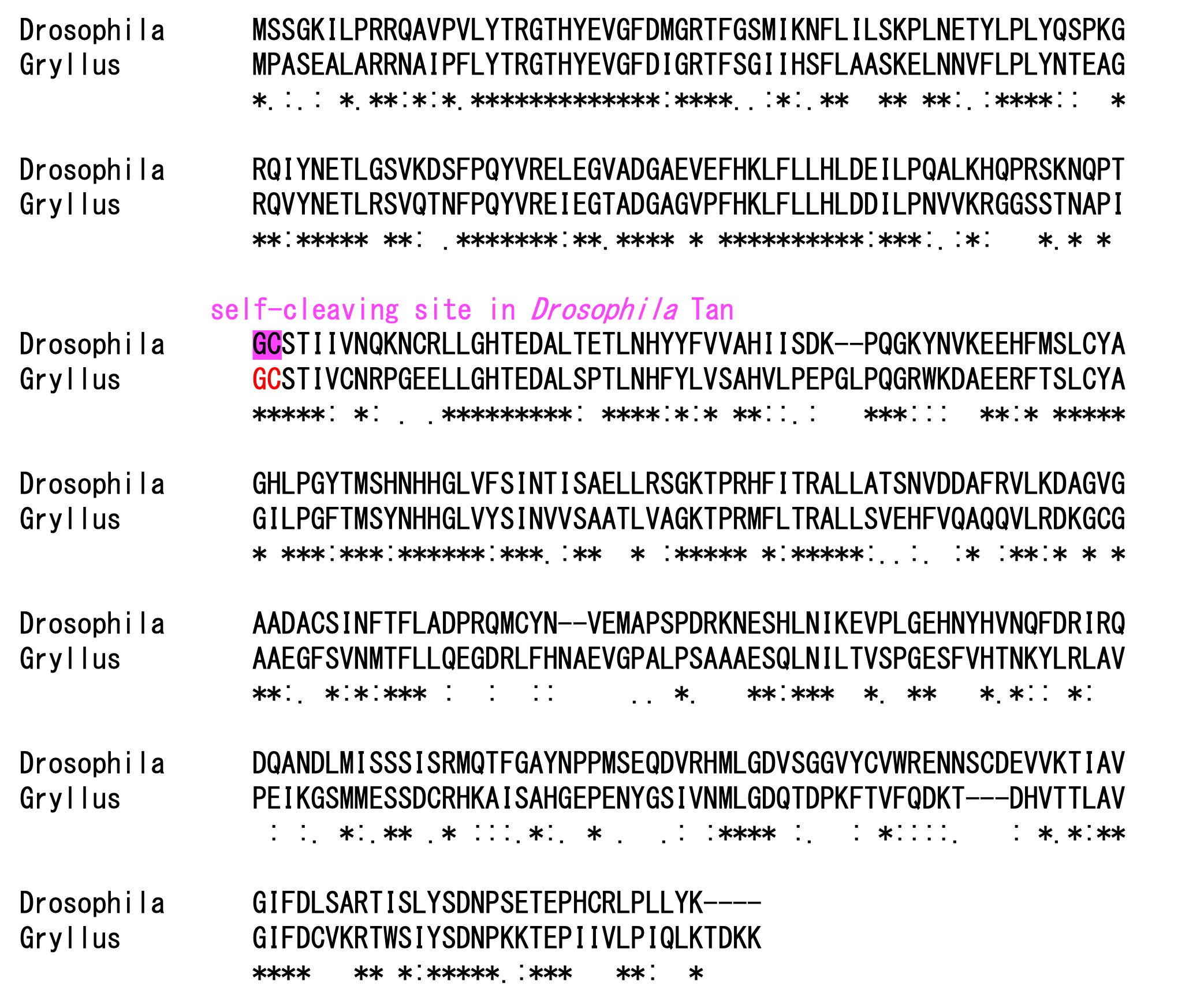

Supplement: S4 Fig — Primary structures of Gb’Tan and D. melanogaseter Tan were aligned using the ClustalW program. The amino acid sequence of the self-cleaving site in D. melanogaseter Tan is conserved in the Gb’Tan protein. (JPG) [file pone.0285934.s004.jpg]

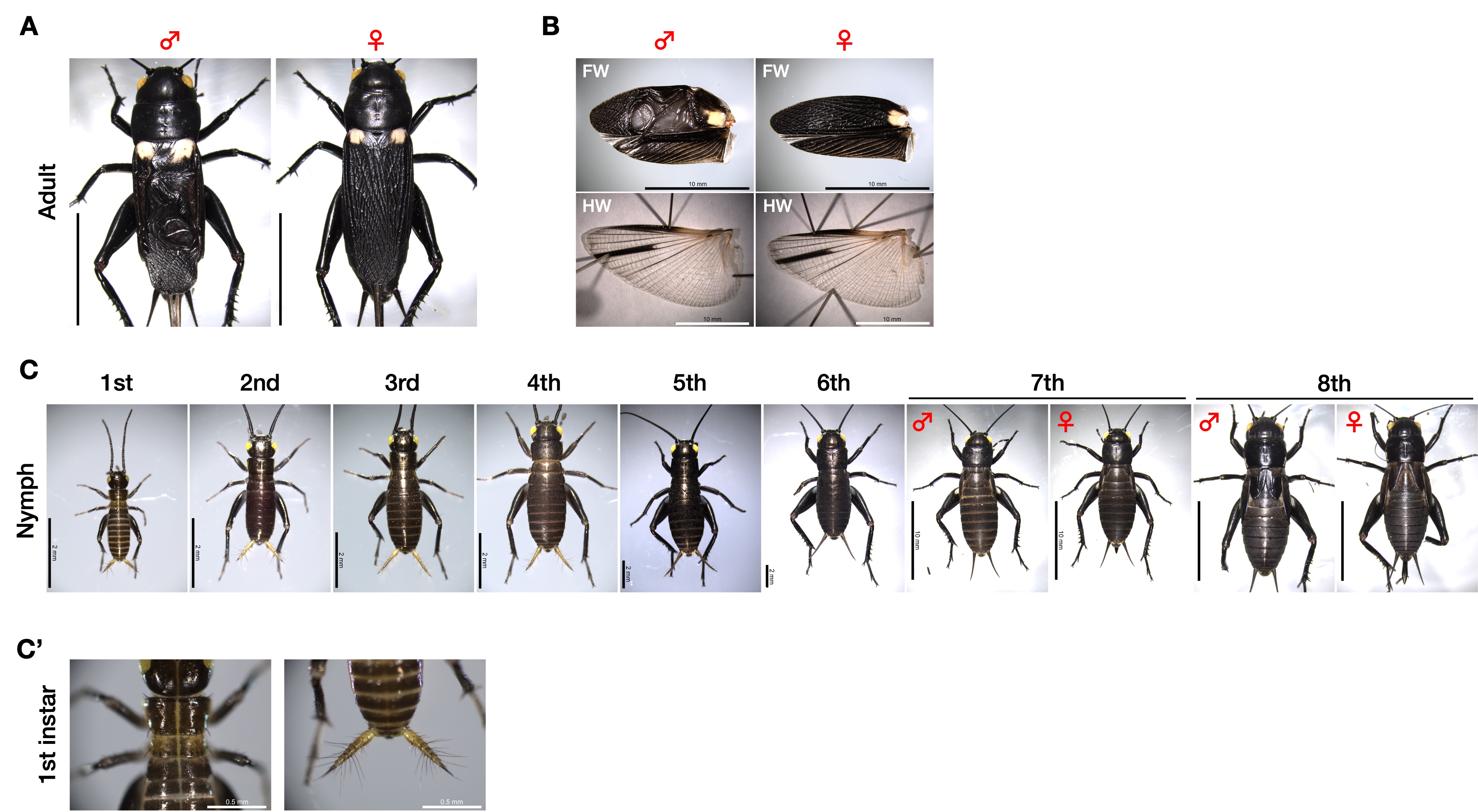

Supplement: S5 Fig — (A) Dorsal views of wild-type and Gb’ebonycr2 mutant adults. (B) Effect of Gb’ebony knockout on the color of adult wings. FW: Forewing, HW: Hind wing. (C) Dorsal views of wild-type and Gb’ebonycr2 mutant nymph stages. (C’) Magnified image of the dorsal side of the thorax and the tail in first instar nymphs. See Fig 5 for a picture of the wild-type. Scale bars: 10 mm in A and B; 2 mm (1st–6th instar nymphs) and 10 mm (7th–8th instar nymphs) in C; 0.5 mm in C’. (JPG) [file pone.0285934.s005.jpg]

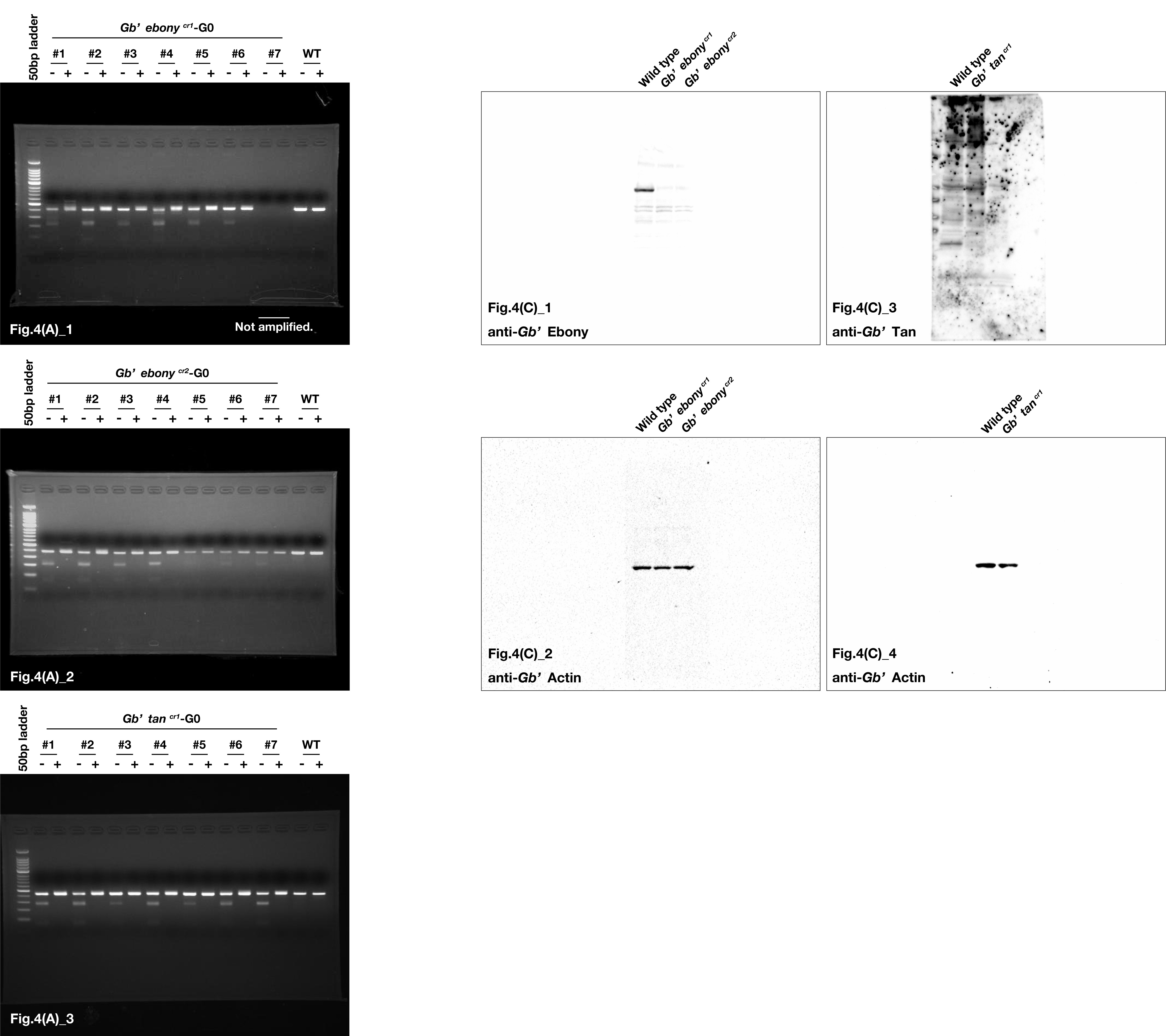

Supplement: S1 Raw images — (JPG) [file pone.0285934.s008.jpg]
