# Supplementary material for: Combinatorial expression of ebony and tan generates body color variation from nymph through adult stages in the cricket, Gryllus bimaculatus
Source: PLoS One. 2023 May 18;18(5):e0285934. doi: 10.1371/journal.pone.0285934 (PMC10194958; doi:10.1371/journal.pone.0285934)
Supplement: S1 Table — (DOCX) [file pone.0285934.s006.docx]

**S1 Table. Primers used in this study.**

| Cloning | Sequence (5’ to 3’) |
| --- | --- |
| *Gb’ebony*-F | ATGGGCTCGATCCCGCAAC |
| *Gb’ebony*-R | TCAGACTACTTTCCAGCAGCATG |
| *Gb’tan*-F | CACACTATGCCTGCCTCTGA |
| *Gb’tan*-R | ATAAAGCACAAAAGCGCACA |
| RT-qPCR |  |
| *Gb’ebony*-qF | GCCCTCGTGCTCTACACATC |
| *Gb’ebony*-qR | CCGACTCCGTATAGGGGAAG |
| *Gb’tan*-qF | TTACTCGCGCCCTTTTAAGT |
| *Gb’tan*-qR | AGGTCCCACTTCTGCATTGT |
| *Gb’actin*-qF | TTGACAATGGATCCGGAATGT |
| *Gb’actin*-qR | AAAACTGCCCTGGGTGCAT |
| Genotyping |  |
| *Gb’ebony ^cr1^*-F | GTGGAGCCCGATGTGTAGAG (-strand) |
| *Gb’ebony ^cr1^*-R | GGACACGCGAAAGCCTAGAAG (-strand) |
| *Gb’ebony ^cr2^*-F | CCGAATGGGACTAGAGCCTTG (-strand) |
| *Gb’ebony ^cr2^*-R | GGTGGAGGAGGAGATGCTG (-strand) |
| *Gb’tan ^cr1^*-F | AGTTCAATCGCGCATCTCTTG |
| *Gb’tan ^cr1^*-R | AACTTTTCGGGTCCACCTTTG |
| PCR of potential-off target site in the *Gb’tan^cr1^* crRNA | |
| Scaffold307-F | GCTTGGCTGGACATCTCTTC |
| Scaffold307-R | CGCTCATCGTTTGTATCGTC |
| Scaffold70-F | GCAATGGGAATAGCAGGAAA |
| Scaffold70-R | GCCATGTTGCCTGGTATTTC |
| Scaffold18-F | GTCGCAAATGCTGAAACGTA |
| Scaffold18-R | ACGAGGATGCGCCATATAAA |
| Subcloning into pET vector |  |
| pET47b-*Gb’ebony*-F | CCGGGTACCAGGATCCGCTCGACCTGGTGACGG |
| pET47b-*Gb’ebony*-R | TACAGAATTCGGATCACCAGCAGCATGTAGCTGT |
| pET47b-*Gb’tan*-F | CCGGGTACCAGGATCTGCCTGCCTCTGAAGCTC |
| pET47b-*Gb’tan*-R | TACAGAATTCGGATCTTACTTCTTGTCTGTTTTCAGC |
