# Supplementary material for: Combinatorial expression of ebony and tan generates body color variation from nymph through adult stages in the cricket, Gryllus bimaculatus
Source: PLoS One. 2023 May 18;18(5):e0285934. doi: 10.1371/journal.pone.0285934 (PMC10194958; doi:10.1371/journal.pone.0285934)
Supplement: S2 Table — Underlined letters indicate the PAM sequence. (DOCX) [file pone.0285934.s007.docx]

**S2 Table. Target sequencies of crRNA.**

Underlined letters indicate the PAM sequence.

|  | Target sequence (5’ to 3’) |
| --- | --- |
| *Gb’ebony*-crRNA1 | CCGTCTCCGCTTCCTCCTGCAGC (-strand) |
| *Gb’ebony*-crRNA2 | CCTTTGGGCACGCCGGTGGAGCC (-strand) |
| *Gb’tan*-crRNA1 | GCAATATGTCCGTGAAATCGAGG |
