# Supplementary material for: Combinatorial expression of ebony and tan generates body color variation from nymph through adult stages in the cricket, Gryllus bimaculatus
Source: PLoS One. 2023 May 18;18(5):e0285934. doi: 10.1371/journal.pone.0285934 (PMC10194958; doi:10.1371/journal.pone.0285934)
Supplement: Attachment — Submitted filename: PONE-D-23-01091_reviewer comments.pdf [file pone.0285934.s009.pdf]

In this manuscript, the melanin synthesis pathway key genes *ebony* and *tan* were identified to regulate the pigmentation and body color patterns in the cricket *Gryllus bimaculatus*, as a model of the hemimetamorphosis insects. Authors found that dynamic alteration in expression levels of *Gb'ebony* and *Gb'tan* in combination was correlated with the body color transition from nymphal stages through adult and knockout of *Gb'ebony* and *Gb'tan* gene resulted darkened systemically and displayed a yellow color in certain areas and stages, respectively. *ebony* and *tan*, as some conservative functional genes, have been studied in many insect species including *Drosophila melanogaster*, *Tribolium casteneum*, *Henosepilachna vigintioctopunctata*, *Oncopeltus fasciatus*, *Bombyx mori*, *Spodoptera litura* and so on. This paper extends the understanding of *ebony* and *tan* to regulate the body color pattern through nymph to adult stage in the hemimetamorphosis insect. However, some critical points should be addressed prior to further its consideration for publication.

Major comments:

1. According to the results of Figure 3, authors showed the expression profiles of *Gb'ebony* and *Gb'tan* transcripts from embryo through adult stages. There were some unexpressed points, such as the D3 of 5th, the D3 of 7th, the D3 of 8th and D3 of adult stage. Please display the time and tissue of the detected samples. And it is better for the expression profiles of genes using the means labeled with different letters indicate significant difference at $P < 0.05$. Two segments of Y axis are more visual to show the low expressed stage in Fig 3A and 3A'.
2. In Figure 4, authors demonstrated that the knockout of *Gb'ebony* and *Gb'tan* genes were successful and the results were solid. But there absented a wild type control of Fig 4A. Authors should display the detail information of the enzyme (Gluide-it Resolvase) including company, place of production, experimental methods and so on. A PCR sequencing of the G0 eggs is better to show the mutant results.
3. From Figure 5 and Figure 6, authors showed many phenotypes of *Gb'ebony* and *Gb'tan* mutants. According to previous reports, the functions of gene *ebony* and *tan* are opposite. The phenotypes of *Gb'ebony* mutants were obvious and convinced. What is different about the homozygous mutants *Gb'ebony*^{cr1} and *Gb'ebony*^{cr2} ?

Why authors chose the homozygous mutants *Gb'ebony*^{cr1} to show the mutant phenotypes? Some other phenotypes could display in the supplemental material. The homozygous mutants *Gb'tan* showed different phenotypes in 7th and 8th. Please explain the related and potential reasons. The western blot of *Gb'tan* gene is not clear and enough to identify the gene function loss of mutant strain of *Gb'tan*. qRT-PCR is a full complement of these results.

4. The sexual dimorphism is ubiquitous in animals and plants, especially in insects. The body color and pigmentation are the typical trait in insect sexual dimorphism. Authors found there are some different phenotypes of body color between male and female adult in *G. bimaculatus*. The hormones and sex determination genes could regulate the expression of *Gb'ebony* and *Gb'tan*. It is an interesting point for researchers.

Others:

1. Line 262, 263 and 398, *Gb'ebony* should be *Gb'ebony*.
2. The format of scale bars should be unified in Figure 5 and 6.
3. It is better to upload the *Gb'ebony* and *Gb'tan* sequences to NCBI and get the GenBank access number.
